# Effects of the Argus II Retinal Prosthesis System on the Quality of Life of Patients With Ultra-Low Vision Due to Retinitis Pigmentosa: Protocol for a Single-Arm, Mixed Methods Study

**DOI:** 10.2196/17436

**Published:** 2021-01-20

**Authors:** Judith White, Laura Knight, Lyndon da Cruz, Paulo E Stanga, Hannah Patrick, Helen Powell, Lee Berry, Kathleen Withers, Grace Carolan-Rees, Timothy L Jackson

**Affiliations:** 1 Cedar Cardiff & Vale University Health Board Cardiff United Kingdom; 2 NIHR Moorfields Eye Hospital London United Kingdom; 3 London Vision Clinic London United Kingdom; 4 Manchester Academic Health Sciences Centre Greater Manchester Mental Health Foundation Trust The University of Manchester Manchester United Kingdom; 5 National Institute for Health and Care Excellence Manchester United Kingdom; 6 Department of Ophthalmology, King's College London London United Kingdom

**Keywords:** patient-reported outcomes, quality of life, qualitative methods, artificial vision, visual function, functional vision, ultra-low vision, low vision, visual function questionnaire

## Abstract

**Background:**

Retinitis pigmentosa is an incurable, degenerative retinal condition causing progressive sight loss, significantly affecting patients’ quality of life. The Argus II Retinal Prosthesis is a surgically implanted medical device that delivers electrical stimulation to the retina. It is intended to produce a form of artificial vision for blind people with severe-to-profound retinitis pigmentosa by stimulating the remaining viable retinal cells to induce visual perception. This study has been initiated by National Health Service England’s Commissioning through Evaluation program and funded through the National Institute of Health Research of the United Kingdom.

**Objective:**

The aim of this study was to assess the effect of the Argus II device on patient’s daily activities and quality of life.

**Methods:**

This protocol is a prospective, single-arm, open-label, mixed methods study on 10 consecutive participants receiving the Argus II device. The patient representatives played an integral role in the design of this study. Eligibility criteria include ultra-low vision in both eyes as a result of end-stage retinitis pigmentosa and a willingness and capacity to complete the postimplantation rehabilitation program. Participants will be interviewed by independent researchers at baseline and 12 months later by using a semistructured, in-depth approach, alongside validated questionnaires (Impact of Vision Impairment-Very Low Vision, 5-level EuroQoL-5 dimensions scale, EuroQoL-visual analog scale, and Hospital Anxiety and Depression Scale) and a bespoke device-related questionnaire, which includes questions about users’ experiences with the procedure, the device, and rehabilitation. The effect of the device on patients’ functional vision and activities of daily living will be assessed by vision rehabilitation specialists using a set of tests measured on an ordinal scale (eg, ability to locate objects and avoid obstacles). Clinical outcomes include full-field stimulus light threshold, square localization, direction of motion, grating visual acuity, Landolt-C, procedural success, and adverse events. Qualitative and quantitative outcomes will be linked in a single database to enable individual participant measures to be considered in toto, comparing baseline to the final review.

**Results:**

This study was approved by the local ethics committee on April 24, 2019 (London-Camberwell St. Giles Research Ethics Committee, reference 19/LO/0429). It has also been approved by the Health Research Authority and Health and Care Research Wales. At the time of protocol writing, Argus II was available for use in the United Kingdom; however, the manufacturer recently withdrew the Argus II device from sale in the United Kingdom. Therefore, the study is not going ahead at this time.

**Conclusions:**

The mixed methods approach provides a rich and in-depth assessment of the effect of the device on participants’ quality of life. Despite the work not going ahead, the publication of this publicly funded protocol is important for researchers planning similar work.

**International Registered Report Identifier (IRRID):**

PRR1-10.2196/17436

## Introduction

### Background

Retinitis pigmentosa is a term for a group of genetically determined degenerative eye conditions that cause progressive loss of retinal photoreceptors. It typically starts with mild loss of peripheral vision, but as the disease advances, vision reduces to a small island of central vision, which may be lost at the end stages of the disease. It is the leading cause of inherited blindness in the United Kingdom, affecting 1 in 4000 people [1]. Although gene therapy has recently been approved for a single genetic subtype [2], there is currently no cure for most forms of retinitis pigmentosa.

### Argus II Retinal Prosthesis System (Argus II)

**Figure 1:**
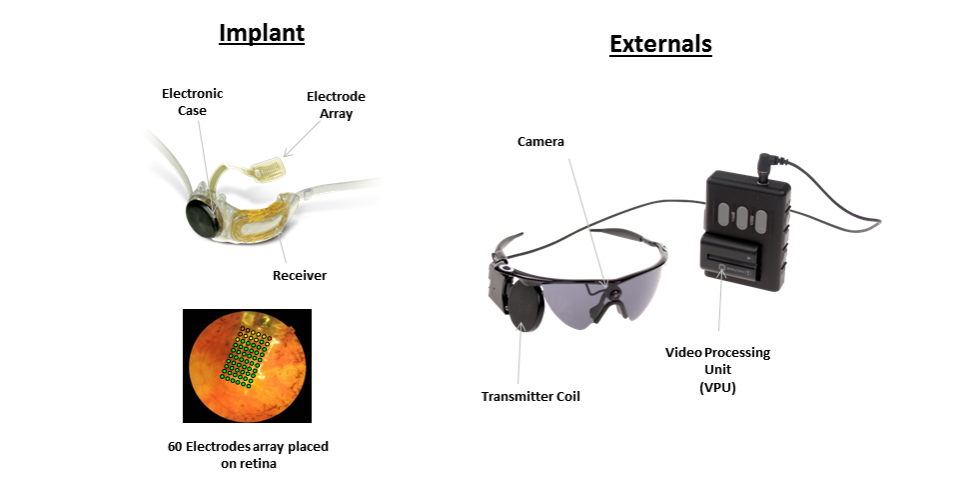
Argus II retinal prosthesis components.

The Argus II Retinal Prosthesis is a medical device that is surgically implanted into only 1 eye and it delivers electrical stimulation to the retina. It is intended to produce a form of artificial vision to blind people with severe-to-profound retinitis pigmentosa by stimulating the remaining viable retinal cells to induce visual perception. It aims to provide functional vision, which enables patients to perceive light, movement, and shapes. The core element of the Argus II system is a spectacle-mounted video camera that records real-time images and a video processing unit that converts the images into data that are wirelessly transmitted to an episcleral receiver unit (Figure 1). This then relays data to the electrode array, which produces electrical impulses that bypass damaged photoreceptors and stimulate the retina’s remaining cells. Visual information is then transmitted by the optic nerve to the brain, creating a visual percept. Through the help of vision rehabilitation professionals, the user learns to interpret these visual patterns to regain some visual function such as perceive light, gain mobility, and identify shapes. Patients require a program of device training coupled with postprocedural rehabilitation to achieve optimal results from the Argus II device [3].

### Current Evidence of the Effects of Argus II

#### Efficacy of Argus II

In 2015, the National Institute for Health and Care Excellence (NICE) published interventional procedure guidance on the insertion of a retinal prosthesis for retinitis pigmentosa [4]. The available evidence was based primarily on 1 prospective multicenter case series of 30 participants [5]. At 5 years after implantation, 12 (40%) patients experienced 24 serious adverse events, including conjunctival erosion, hypotony, conjunctival dehiscence, and endophthalmitis. By 5 years, 2 (6.7%) Argus II devices failed. In both cases, the reason was progressive loss of the radiofrequency link between the external antenna and the implant.

#### Effect on Visual Function

There is evidence that Argus II produces improvements in visual function and observer-rated functional vision tasks, albeit in a small number of patients in a clinic environment, when comparing the device in its “ON” and “OFF” position [5-8]. Functional vision was assessed using the Functional Low-Vision Observer Rated Assessment tool, a multicomponent questionnaire consisting of 35 observer-rated tasks organized into 4 domains: visual orientation, visual mobility, daily life, and interaction with others. On average, patients were able to complete 24 of the 35 tasks (69%) more easily with the Argus II device switched ON than when the Argus II device was switched OFF; 2 tasks (6%) were harder to complete on average, and 9 tasks (26%) showed no significant change between the ON and OFF positions (the authors did not report the number of patients who showed a change) [7]. Using orientation and mobility tasks (“find the door” and “follow the white line”), all patients performed better with the device switched ON than when the device was switched OFF at all time points (28 patients at 1, 2, and 3 years; 22 patients at 4 years; and 20 patients at 5 years) [5]. Patients performed better at 3 real-world functional vision tasks: in a sock sorting task (bare table), 21 of the 28 patients (75%) performed better with the system ON; in the sidewalk tracking task, 18 of the 27 patients (67%) performed better with the system ON; and in the walking direction discrimination test, 18 of the 27 subjects (67%) performed above chance with the system ON and 6 (22%) did so with system OFF [8].

#### Effect on Quality of Life

Currently, there is limited evidence on the effect of Argus II on patients’ quality of life (QoL), with 1 study reporting scores from a vision-specific, multi-attribute utility instrument. The Vision and Quality of Life Index (VisQoL) consists of 6 dimensions (injury, life, roles, assistance, activity, and friendship) and was completed by patients before and after receiving Argus II at 3, 6, 12, 18, 24, and 36 months [9]. Composite VisQoL scores at follow-up (presumably all follow-up time points although the authors do not specify this) showed no statistically significant change from the baseline. In 3 of the 6 VisQoL dimensions (injury, life, and roles), there was a significant and lasting improvement after implantation with Argus II in patients whose blindness was affecting their QoL at baseline [9]. No published qualitative studies have been carried out on patients who have received Argus II; a recent report by Health Quality Ontario reports narrative accounts but does not report a full methodology, and this work is not published in a peer-reviewed journal [10].

NICE’s Interventional Procedures Guidance (IPG519) [4] has recommended further research focusing on “...the impact on quality of life and activities of day-to-day living, and durability of implants.” The committee “wanted evidence that any changes in metrics of vision result in improved QoL and activities of daily living.” These recommendations have been reflected in National Health Service (NHS) England’s Clinical Commissioning Policy on Argus II retinal prosthesis for retinitis pigmentosa [11].

This study was initiated by NHS England as part of Commissioning through Evaluation, which is part of their Evaluative Commissioning Program. Commissioning through Evaluation enables a limited number of patients to access treatments that are not routinely funded by the NHS, but nonetheless show significant promise for the future, while new clinical and patient experience data are collected within a formal evaluation program. This study was commissioned to develop evidence on the effect of Argus II on patients’ QoL, in order to inform the NHS England commissioning policy for the procedure.

## Methods

### Aim, Design, and Setting of the Study

This study aims to assess the impact of the Argus II Retinal Prosthesis System (Second Sight) on the QoL of participants with ultra-low vision as a result of retinitis pigmentosa. This protocol is for a single-arm, prospective, open-label, mixed methods, multicenter, before versus after study on 10 participants. All participants will receive Argus II as part of the research study and will be required to take part in the rehabilitation program following surgery. A mixed methods approach is required because neither qualitative nor quantitative methods alone would support the in-depth analyses of the effect of Argus II on participants’ QoL. The study will take place in several settings. The retinal prosthesis will be implanted and fitted in a specialist eye hospital, where clinical examinations will also take place. The vision rehabilitation training and assessments will take place in an outpatient clinic and in participants’ homes. Recruitment is expected to take 12 months, and each participant will be followed up for 12 months.

### Study Population

The population will be adults with ultra-low vision in both eyes as a result of retinitis pigmentosa. Participants must have severe-to-profound outer retinal degeneration (not including age-related macular degeneration), with some residual light perception or with retinal response to electrical stimulation and with history of useful form vision. Eligible participants must provide consent for the procedure, a program of rehabilitation, clinical data collection, and agree to take part in qualitative interviews and questionnaire completion (administered and analyzed by independent researchers from an NHS research center, Cedar, Cardiff & Vale University Health Board).

The exclusion criteria for the patients would be as follows: (1) ocular diseases or conditions that could prevent Argus II from working, (2) ocular structures or abnormalities that could prevent the successful implantation of the Argus II implant or adequate healing following surgery, (3) ocular diseases or conditions (other than cataracts) that prevent adequate visualization of the inner structures of the eye (eg, corneal opacity), (4) predisposition to eye rubbing, (5) inability to tolerate general anesthesia or the recommended antibiotic and steroid regimen associated with the implantation surgery, and (6) any disease or condition that prevents understanding or communication of informed consent, study demands, testing protocols, and qualitative interviews.

Eligible participants will be provided with an information pack at or before their initial study visit and the clinical investigators will read all of the written information to the participant. Participants will be offered an audio recording and electronic version of the participant information sheet and informed consent form.

### Sampling

Ten participants will be recruited over a 1-year period to take part in the study. The recruitment rate is based on UK recruitment experience during a previous clinical trial of the Argus II implant [5]. The procedure is a highly specialized treatment and large numbers of participants would be difficult to recruit. In addition, NHS England (the commissioning body) recognized that approximately 10 people per year would be eligible for the Argus II procedure [11]. Consecutive participants who meet the eligibility criteria will be invited to take part. The choice of a sample size of 10 participants is also based on pragmatic considerations of time and budget (ie, convenience). The aim will be to reach or at least approach data saturation by the end of follow-up of the tenth participant. This approach is supported by the work of Francis et al (2010) [12], whereby an initial analysis sample of 10 interviews was chosen. We accept that limiting our sample size to 10 may not identify every key theme, but we expect to identify those most important to the majority of the participants.

If a participant withdraws prior to receiving the implant, they will be replaced by another participant. If a participant withdraws after the implant is fitted, they will retain the device but will no longer be required to complete any subsequent study visits.

### Study Device

The Argus II Retinal Prosthesis System is an active implantable medical device. It received a CE mark in 2011. It is intended to “provide electrical stimulation of the retina to induce visual perception in blind individuals” (from Second Sight Instructions for Use). The Argus II system has 2 key groups of components (Figure 1): (1) the external components comprising a small video camera mounted on a pair of spectacles, which is connected via a cable to a video processing unit worn on a belt or a shoulder strap. The video camera captures images, which are converted by the video processing unit into stimulation commands. These are wirelessly transmitted to the internal components and (2) the implanted components, which include an episcleral receiver unit, electronics, and an electrode array that are surgically implanted in and around the eye. The array is attached to the retina over the macula. When the system is functioning, data from the video processing unit is received by the subconjunctival receiver unit, which communicates directly with the electrode array through a permanent sclerotomy. The electrodes emit electrical impulses to stimulate the sensory neurons of the surviving retinal cells, which send visual information to the brain via the optic nerve.

### Schedule of the Procedures and Assessments

#### Insertion and Fitting of the Retinal Prosthesis

Argus II is intended to be implanted in a single eye and should be implanted in the worse-seeing eye. If both eyes have equivalent residual vision and are equally suitable for implantation, the participant’s preference for the implanted eye should be respected (from Second Sight Instructions for Use). Insertion of the implanted device components is performed with the participant under general anesthesia, usually in a single procedure taking several hours. The surgeon performs core and peripheral vitrectomies, followed by dissection of any retinal membrane in the area where the electrode array will be placed. The electrode array is inserted through a superotemporal sclerotomy and secured on the retina using a retinal tack. It is connected to the receiver unit by a cable that penetrates the sclera in the pars plana. This cable is sutured flat against the external sclera at the point of exit and is covered with a piece of donor sclera (Tutoplast) to avoid exposure and erosion. Intraoperative adverse events and complications will be recorded as part of this study.

Following implantation (typically 1 week after surgery), the device will be customized by a fitting technician in an outpatient clinic where the video processing unit is programmed specifically for use by the subject (Table 1). The basic fitting process involves implant diagnostics (eg, electrode impedance measurements), array scanning (ie, determination of stimulation thresholds for each single electrode), and the creation of one or more video configuration files, which contain the information of how the video signal is mapped to the electrical signal of the electrode array.

**Table 1:** Schedule of the study procedures and assessments.

Role, study procedure/assessment			–60 to –1 days	Day 0	Day 1	Week 1	Week 2	Month 1	Month 3	Month 6	Month 12
**Clinical teams**											
	Enrolment/consent		✓								
	Patient history		✓								
	Clinical examinations including eye examination, retinal photography, visual acuity		✓		✓	✓	✓	✓	✓	✓	✓
			✓					✓	✓	✓	✓
	Functional vision, including square localization test, direction of motion test		✓						✓	✓	✓
	Adverse events (including relatedness to device)			✓	✓	✓	✓	✓	✓	✓	✓
	Resource use		✓	✓	✓	✓	✓	✓	✓	✓	✓
**Researchers independent of the clinical team**											
	Semistructured interviews		✓^a^								✓
	**Quantitative QoL outcomes**										
		IVI-VLV^b^	✓							✓	✓
		EQ-5D-5L and EQ-VAS^c^	✓							✓	✓
		HADS^d^	✓							✓	✓
		Bespoke device-related questionnaire^e^							✓	✓	✓
**Rehabilitation staff**											
	Training and visual rehabilitation reports							Sessions in clinic and at patient’s home			
	Visual function tests		✓								✓

^a^5-30 days prior to implantation.

^b^Changes in vision-related QoL (quality of life) will be measured using the IVI-VLV (Impact of Vision Impairment-Very Low Vision) questionnaire.

^c^Changes in general health-related QoL will be measured using the EQ-5D-5L (5-level EuroQoL 5-dimension scale) and EQ-VAS (EuroQoL visual analog scale) questionnaires.

^d^Changes in symptoms of anxiety and depression will be measured using the HADS (Hospital Anxiety and Depression Scale) questionnaire.

^e^The overall impact of Argus II as well as pain/discomfort from the device, perceived complications, satisfaction with results of procedure, and satisfaction with rehabilitation will be measured using a bespoke questionnaire.

#### Clinical Follow-up

Clinical follow-up visits are planned at 1 day postimplantation, at weeks 1 and 2, and then at 1, 3, 6, and 12 months after the implantation (these visits will be modified in line with local routine practice, Table 1). In the event that COVID-19 precautions are in place, some clinical follow-ups may be carried out remotely where the treating clinician deems it is appropriate. Most of the visual function tests and measures will be conducted in both the implanted and fellow eye to provide data on the natural course of the participants’ vision loss and as a control for measurements of visual function. Testing will also compare visual function in the study eye with the device ON versus that in the study eye with the device OFF. The number of follow-up visits will be recorded. Unscheduled visits such as those required to address potential adverse events will also be recorded.

#### Baseline Assessments

At the baseline visit (60 days to 1 day prior to the implantation procedure and after consent has been given), clinical data will be recorded in a clinic setting, including complete eye examination, medical evaluation, retinal photography, and optical coherence tomography, ultrasound A-scan and B-scan, photographic flash test (using a camera flash to assess whether a patient has perception of light; the flash is set off in front of the patient’s eye to confirm any residual response to light), visual acuity tests, as well as a psychosocial evaluation in order to ensure that the subject has realistic expectations about the system (Table 1).

#### Participant Interviews and Questionnaires

Participants will be interviewed by independent researchers at baseline (approximately 5-30 days prior to the implantation procedure) and at 12 months follow-up (±1 month). More frequent in-person interviews were ruled out as being overly burdensome to patients. Interviews will be semistructured (using a topic guide, Multimedia Appendix 1) with opportunities for unstructured conversation. Structure is required to ensure that certain areas of interest are explored during the interviews, but the approach will be kept flexible enough to explore the impact of the device on all aspects of participants’ lives and allow for unexpected findings. Interviews will be preferentially carried out in-person at participants’ homes. Where this is not possible (such as in cases where COVID-19 precautions are in place), interviews can be carried out by phone. An interview/prompt guide will be finalized and piloted on participants who have received the Argus II device already. Each researcher will use the same prompts to guide their interview and to cover the same topic areas. A series of questionnaires will be read aloud to participants by independent researchers in person or by telephone at 6 and 12 months follow-up (the bespoke device-related questionnaire will also be administered at 3-month follow-up) (Table 1 and Table 2).

The questionnaires will be Impact of Vision Impairment-Very Low Vision (IVI-VLV) [13], 5-level EuroQoL-5 dimension (EQ-5D-5L) scale, EuroQoL visual analog scale (EQ-VAS) [14], Hospital Anxiety and Depression Scale (HADS) [15], and bespoke device-related questionnaire to obtain structured responses from participants to questions related to the device and the participant experience.

**Table 2:** Qualitative and quantitative outcome measures.

Outcomes		Qualitative measures	Quantitative measures
**Participant-reported outcomes**			
	Impact of device on quality of life	Semistructured/unstructured interviews (in-person or by phone) at baseline and 12 months	Vision-related QoL^a^ using the Impact of Vision Impairment-Very Low Vision validated questionnaire at baseline, 6 months, and 12 months. General health-related QoL measured using EQ-5D-5L^b^ and EQ-VAS^c^ questionnaires at baseline, 6 months, and 12 months.
	Device-related experience	Semistructured/unstructured interviews (in-person or by phone) at baseline and 12 months	Bespoke questionnaire, which includes questions about pain/discomfort from the device, perceived complications, satisfaction with the results of the procedure, satisfaction with rehabilitation, and overall impact of the device at 3 months, 6 months, and 12 months
	Psychological measures	N/A^d^	HADS^e^-validated questionnaire at baseline, 6 months, and 12 months
**Outcomes reported by rehabilitation staff**			
	Delivery/implementation of rehabilitation and device training	Semistructured interviews with rehabilitation staff to record rehabilitation and training strategies, fidelity of delivery, satisfaction with rehabilitation and training program, barriers and facilitators after 5 patients received Argus II and at the end of the study	N/A
	Impact of device on activities of daily living	Short “session report” by rehabilitation staff, which records number of visits, length of visit, and “training” strategies delivered at each visit	Semiquantitative: Visual function tests assessed by rehabilitation staff and recorded on an ordinal scale at baseline and 12 months
**Clinical outcomes**			
	Safety outcomes	N/A	Procedural success All-cause adverse events and serious adverse events Management and outcome of adverse events Device explantation rate
	Visual function	N/A	Full-field stimulus light threshold Square localization test Direction of motion test Grating visual acuity test Landolt-C test
	Resource use	N/A	Proforma for clinical teams, including number of consultations, staff grade, length of procedure, additional interventions, adverse event management Rehabilitation use costed separately from device-related resource

^a^QoL: quality of life.

^b^EQ-5D-5L: 5-level EuroQoL 5-dimension scale.

^c^EQ-VAS: EuroQoL visual analog scale.

^d^N/A: not applicable.

^e^HADS: Hospital Anxiety and Depression Scale.

The IVI-VLV questionnaire, developed by Finger et al [13] in 2014, has been chosen as a vision-related QoL outcome in this study to assess the true effect of the Argus II device on participants’ QoL. The IVI-VLV is a self-rated 28-item questionnaire for use in participants with severe vision loss. Questions are split into 2 subscales: (1) 12 items in the emotional well-being subscale and (2) 16 items in the activities of daily living, mobility, and safety subscale. The tool has been validated and is suitable for use as an outcome measure in trials attempting sight restoration [13]. It can differentiate between different levels of vision-related QoL in participants, and its results are unaffected by levels of self-perceived general and mental health. This was chosen over VisQoL used in a previous Argus II study [9] because the latter is a much shorter (6-item) vision-related utility instrument for the health economic evaluation of eye care and rehabilitation programs rather than a tool to obtain in-depth patient-focused feedback. A generic tool, EQ-5D-5L, is proposed to collect non–disease-specific measures by using a well-established and cross-specialty questionnaire. The EQ-5D-5L comprises the widely used global health questionnaire that provides a simple descriptive profile and a single index value for health status [14].

HADS will be used in the study to examine the effect of Argus II on depression and anxiety. The HADS is a validated and widely used self-rating scale that measures anxiety and depression in both hospital and community settings [15]. This is an important outcome, as mental health issues are prevalent in this patient group. The questionnaire is composed of 14 items (7 for the anxiety subscale and 7 for depression subscale) and can be answered within 2-5 minutes.

A short bespoke (nonvalidated) questionnaire is proposed to provide a structured survey response to questions related to the device and the participant experience. Likert scales or check boxes will be used to assess the following: frequency and duration of device use in the past week, tasks performed with help of the device, pain/discomfort due to the device, perceived complications from the device/procedure, satisfaction with the results of the procedure, and overall effect of the device. The device-related questionnaire will also include a free-text section to capture other information that participants consider important.

#### Vision Rehabilitation Assessments

The impact of the device on participants’ functional vision and activities of daily living will be assessed by vision rehabilitation specialists using a set of visual function tests recorded on an ordinal scale at baseline and 12 months (Table 1 and Table 2). Vision rehabilitation specialists will complete a short “session report,” which describes the actual “training” strategies delivered to the participants, the duration, and the setting of the visit. The proforma will record whether the vision rehabilitation specialists delivered the rehabilitation program as planned and any deviation or adaptations to the planned intervention. Independent researchers will carry out semistructured interviews with vision rehabilitation specialist staff at the study halfway point (after 5 participants have received Argus II) and then at the study end. These interviews will record rehabilitation and training strategies, fidelity of delivery, satisfaction, context, barriers, and facilitators. Once subjects have completed the 1-year follow-up, they will continue to use the device and will be followed per the standard of care (ie, follow-up visits every 12 months).

#### Outcomes

The outcome measures are described in Table 2.

### Data Management and Analysis

#### Qualitative Data

Audio recordings of each interview will be transcribed verbatim into a standard word processing document by independent researchers. All potentially identifiable participant data will be deidentified in the transcript. Transcripts will be imported and coded using computer-assisted qualitative data analysis software (NVivo, QSR International). Analysis of the qualitative data will use a mainly iterative-inductive approach, whereby emergent categories and ideas are generated based on specific observations and measures, rather than *a priori* concepts. This approach is committed to retaining diversity and complexity in the analysis. Furthermore, we aim to respect the uniqueness of individual cases as well as identifying comparative themes and patterns. Inductive thematic analysis (using elements of Grounded Theory as a set of procedures for coding data) will be used to identify themes in the data and to combine them to achieve a coherent interpretation of how the Argus II device affects participants’ QoL.

#### Quantitative Data

Clinical data collected at the treating sites will be transferred to a secure electronic study database. Responses from the participants to questionnaires will be recorded on paper copies by independent researchers and then transferred to the study database. Data will be reidentified to enable linkage with qualitative data to provide richness. The aim of this mixed methods approach will be to investigate not only “did the implant improve vision and quality of life” (from quantitative data sources) but also “how” and “why” and most importantly the patients’ own perception of the impact on their QoL (from qualitative data). For instance, questionnaires may point toward measurable improvements between baseline and follow-up in vision and QoL; when linked to qualitative interview data, we can better understand the factors that influenced this result such as previous patient strategies for dealing with vision loss, existence of mental health issues, home living arrangements and support network, perception of the impact of the device, perception of rehabilitation support, and patient engagement with rehabilitation service. Individual participant measures will be presented in a case report format where appropriate, with a focus on changes from baseline to follow-up. Descriptive statistics will be generated across all measures. No statistical comparisons will be carried out due to the small sample size.

#### Adverse Events

All participants who have been exposed to the study treatment will be evaluated for adverse events at each visit. All adverse events, regardless of the severity or seriousness and whether they are ascribed to the study treatment, will be recorded in the source documents and case report form by using standard medical terminology and coded using the Medical Dictionary for Regulatory Activities Preferred Terms. The adverse event severity, action taken, outcome, follow-up, and relatedness to study device will be recorded and escalated appropriately.

### Patient and Public Involvement

Two patient representatives sat on the Steering Group for this study, which had met regularly since the project’s inception. Both have been treated with the Argus II device in a previous study and they provided valuable insights to the research teams regarding the acceptability of the planned research and how best to design the study from a patient perspective. Both patient representatives reviewed the participant information sheet and informed consent form and provided feedback.

### Ethical Considerations

This study is a clinical trial of a CE-marked active implantable medical device being used as per the manufacturer’s intended purpose. The manufacturer’s instructions for use will be followed at all times. Previous studies suggest that there is a material risk of adverse events associated with the implantation of Argus II. Clinical teams and rehabilitation specialists will monitor participants closely following implantation of the device to ensure that adverse events are identified and treated promptly. Risks to participant data confidentiality will be mitigated through transfer of only deidentified participant data between treatment sites (questionnaire and interview data will be deidentified). Protocol adherence will be monitored at both clinical sites by the sponsor (King’s College London). This study has been reviewed by and given favorable ethical opinion by London Camberwell St. Giles Research Ethics Committee (REC Reference: 19/LO/0429). It has also been approved by the Health Research Authority and Health and Care Research Wales. Written informed consent will be obtained from eligible patients prior to enrolment.

## Results

This study was approved by the local ethics committee on April 24, 2019 (London-Camberwell St. Giles Research Ethics Committee, reference 19/LO/0429). At the time of protocol writing, Argus II was available for use in the United Kingdom; however, during preparation for study initiation, the manufacturer (Second Sight) suspended worldwide production of the device, resulting in the suspension of commercialization in the United Kingdom and other international markets (to direct resources to development of the Orion cortical implant). We felt it important to publish this protocol so that the publicly funded work to develop the protocol is made publicly available and that researchers planning similar research on other retinal or low vision devices could learn from our work.

## Discussion

### Overview

Retinitis pigmentosa is a disabling disease, which currently has no cure. Insertion of a retinal prosthesis offers a potentially important treatment by restoring perception of light, movement, and shapes, but the effect of this relatively basic visual function on QoL is unknown. This study was part of NHS England’s Commissioning through Evaluation program, which is part of its Evaluative Commissioning Program. Commissioning through Evaluation enables a limited number of patients to access treatments that are not routinely funded by the NHS, but nonetheless show significant promise for the future, while new clinical and patient experience data are collected within a formal evaluation program. NHS England’s clinical commissioning policy states that there is not sufficient evidence to support the routine commissioning of Argus II for retinitis pigmentosa and that, based on the recommendation by the Rare Diseases Advisory group, further evaluation is required before making the treatment available. In addition, NICE IPG519 recommends further research on this technology, including outcomes on the impact on QoL and activities of day-to-day living and durability of implants. The proposed Commissioning through Evaluation study has been designed to fill this evidence gap by providing first-hand narrative accounts of patients about the effects on their daily activities and QoL before and after receiving a prosthesis.

### Strengths and Limitations

The key strength of this study is the mixed method design, which used both qualitative and quantitative outcomes to provide a rich and in-depth evaluation of the effect of the Argus II device on the quality of patients’ lives. Patients previously treated with Argus II have sat on the Steering Group and have been integral to the study design and production of patient-facing documentation and assessment tools. The involvement of independent researchers to gather qualitative data from patients and vision rehabilitation specialists lends credibility to the research. Furthermore, collecting data on the provision of a rehabilitation service adds a unique and important insight to this research on a complex intervention.

The main limitation of this study is the small sample size. Only 30 patients have previously received this technology in the United Kingdom in a research context. We accept that limiting our sample size to 10 may not identify every key theme when interviewing participants, but we expect to identify those most important to the majority of the participants. Purposive sampling, which aims to select the most information-rich cases (eg, extreme sampling or maximum variation sampling), is not appropriate for this study because of the ethical problems of denying the Argus II intervention to eligible participants.

## Appendix

Multimedia Appendix 1 Interview topic guide.

## Abbreviations

EQ-5D-5L: 5-level EuroQoL 5-dimension scale
EQ-VAS: EuroQoL visual analog scale
IVI-VLV: Impact of Vision Impairment-Very Low Vision
NHS: National Health Service
NICE: National Institute for Health and Care Excellence
QoL: quality of life
VisQoL: Vision and Quality of Life Index
