# Supplementary material for: Effects of the Argus II Retinal Prosthesis System on the Quality of Life of Patients With Ultra-Low Vision Due to Retinitis Pigmentosa: Protocol for a Single-Arm, Mixed Methods Study
Source: JMIR Res Protoc. 2021 Jan 20;10(1):e17436. doi: 10.2196/17436 (PMC7857949; doi:10.2196/17436)
Supplement: Multimedia Appendix 1 [file resprot_v10i1e17436_app1.docx]

**Supplemental material**

**Interview topic guide - Pre-procedure (baseline)**

*This guide is designed as a reminder of areas for discussion. Items should be used as prompts only. This is not a survey. Provide the participant with an overview of the purpose of interview, e.g. to understand how their visual impairment impacts on their life, and to understand their expectations regarding the retinal implant procedure with the Argus II device. Remind participants of your intention to record the audio.*

**Key areas & non-compulsory prompts**

**Tell me about your HISTORY of visual impairment**

- diagnosis
- change over time
- past and present levels of visual impairment

**Tell me about how your visual impairment IMPACTS on your DAILY LIVING**

- positives & negatives
- independent living
- daily activities
- socialising
- employment

**Tell me about how your visual impairment IMPACTS on HOW YOU FEEL**

- identity
- confidence or self-esteem
- well-being
- self-image
- relationships

**Tell me about the ways you MANAGE your visual impairment**

- vision rehabilitation
- assistive devices or mobility aids
- support from friends and family
- support from professionals

**Tell me about the EXPECTATIONS you have for the PROCEDURE and DEVICE**

- knowledge and reasons for participation
- expected changes
- hopes and goals for future
- concerns or fears
- optimism/pessimism

**Interview topic guide - Post-procedure (follow-up)**

*This guide is designed as a reminder of areas for discussion. Items should be used as prompts only. This is not a survey. Provide the participant with an overview of the purpose of interview, e.g. to understand how the Argus II device has impacted on their lives. Remind participants of your intention to record the audio.*

*Refer to notes/transcripts from baseline interview if relevant.*

**Key areas & non-compulsory prompts**

**Tell me about your EXPERIENCES with having the DEVICE FITTED**

- positive and negative emotions
- time in hospital
- recovery
- pain
- complications
- support from professionals

**Tell me about your INITIAL EXPERIENCES and LEARNING TO USE the DEVICE**

- rehabilitation/ learning
- who? professionals, friends, family
- duration
- feelings about learning
- difficulty
- practice
- impact on use of other techniques

**Tell me about your ongoing EXPERIENCES with REHABILITION and USING the DEVICE**

- positive and negative emotions
- Who?

**Tell me about how you USE the DEVICE in your daily life**

- setting (home, work, unfamiliar settings, inside, outside)
- tasks
- skills
- capabilities
- enhance/interfere with existing skills
- most helpful in situations
- least helpful in situations

**Tell me about how the DEVICE has IMPACTED your DAILY LIVING**

- what do you like/dislike about the device?
- things you can do now which you couldn’t before
- independent living
- daily activities
- socialising
- employment

**Tell me about changes in how you FEEL since getting the DEVICE**

- identity
- independence
- confidence or self-esteem
- well-being
- self-image
- relationships

**Tell me about how your EXPECTATIONS from 1 year ago have or have not been met**

- achievements/rewards
- disappointments/regrets
- surprises
- management of expectations
- accuracy of information provided

**Tell me about your GOALS FOR THE FUTURE in relation to your visual impairment**

- continue using the system
- other aides
- tasks
- practice
- support
